# Expert Panel Recommendations for Use of Standardized Glucose Reporting System Based on Standardized Glucometrics Plus Visual Ambulatory Glucose Profile (AGP) Data in Clinical Practice

**DOI:** 10.3389/fendo.2021.663222

**Published:** 2022-01-24

**Authors:** Selcuk Dagdelen, Oguzhan Deyneli, Nevin Dinccag, Hasan Ilkova, Zeynep Osar Siva, Ilhan Yetkin, Temel Yilmaz

**Affiliations:** ^1^ Department of Endocrinology and Metabolism, Hacettepe University Faculty of Medicine, Ankara, Turkey; ^2^ Department of Endocrinology and Metabolism, Koc University Faculty of Medicine, Istanbul, Turkey; ^3^ Department of Endocrinology and Metabolism, Istanbul University Istanbul Faculty of Medicine, Istanbul, Turkey; ^4^ Department of Endocrinology and Metabolism, Istanbul University Cerrahpasa Faculty of Medicine, Istanbul, Turkey; ^5^ Department of Endocrinology and Metabolism, Gazi University Faculty of Medicine, Ankara, Turkey; ^6^ Department of Endocrinology and Metabolism, Florence Nightingale Hospital, Istanbul, Turkey

**Keywords:** diabetes care, continuous glucose monitoring, ambulatory glucose profile, expert opinion, clinical utility, algorithm

## Abstract

This expert panel of diabetes specialists aimed to provide guidance to healthcare providers on the best practice in the use of innovative continuous glucose monitoring (CGM) techniques through a practical and implementable document that specifically addresses the rationale for and also analysis and interpretation of the new standardized glucose reporting system based on standardized CGM metrics and visual ambulatory glucose profile (AGP) data. This guidance document presents recommendations and a useful algorithm for the use of a standardized glucose reporting system in the routine diabetes care setting.

## Introduction

Limitations of glycated hemoglobin (HbA1c) *per se*, underutilization of continuous glucose monitoring (CGM), and lack of an easy to interpret and standardized system for glucose reporting are considered among the key contributors to continued suboptimal glycemic control in diabetes patients despite advances in therapeutics (1).

The main drawbacks of self-monitoring blood glucose (SMBG) systems include inadequate patient compliance to intermittent capillary sampling, unreliability of patient-recorded data, inability to capture and store large amounts of glycemic data including hypoglycemic episodes for extended periods of time (2). In this regard, as strongly advocated by recent consensus statements, the use of CGM *via* a standardized metric reporting, using the ambulatory glucose profile (AGP) for data visualization, is considered a part of the evolving standard of diabetes care, supplementing periodic HbA1c testing in addition to accurate evaluation of glycemic variability, and the identification of nocturnal hypoglycemic episodes (1–6).

CGM, involving the real-time CGM (rtCGM) and the flash glucose monitoring (FGM) also known as intermittently scanned CGM (isCGM), has become an increasingly used method with technical improvements over time (i.e., in sensor accuracy, convenience and ease of use, and reimbursement conditions) (7). However, despite the increased CGM adoption in insulin-requiring diabetes care as recognized by national and international medical organizations in many countries, successful utilization of CGM data in real-life diabetes care remains relatively low (1, 7).

The recent innovations in sensor technology combined with standardization and simplification of the analysis of glucose data *via* AGP enable an improved method for retrospective analysis of rtCGM/FGM profiles (1, 8). However, it may remain underutilized in clinical practice due to concerns regarding the complexity and inconvenience of CGM use among physicians and patients and also the clinician’s reluctance due to lack of experience in interpreting CGM data.

This expert panel of diabetes specialists therefore aimed to provide guidance to healthcare providers on the best practice in the use of innovative CGM techniques through a practical and implementable document that specifically addresses the analysis and interpretation of the new standardized glucose reporting system based on standardized CGM metrics and visual AGP data, and to provide consensus recommendations and a practical algorithm for the potential use of this reporting system in the routine diabetes care setting.

## Methods

The present expert panel involved seven diabetes specialists who are key opinion leaders with at least 15 years of experience in dealing with diabetes in Turkey. The panel critically analyzed recommendations from existing guidelines, consensus statements and data from systematic reviews, meta-analyses and literature review of articles published on glycemic control and blood glucose testing in type 1 (T1DM) and type 2 (T2DM) patient populations and agreed on a series of statements supported by scientific evidence and expert clinical opinions to assist healthcare providers on the best practice in analysis and interpretation of the new standardized glucose reporting system based on standardized CGM metrics plus AGP data in diabetes care.

Therefore the main areas addressed by this consensus document include a) an overview of glycemic parameters and diabetes-related complications, b) methods for glucose testing/monitoring (limitations of HbA1c and SBMG, CGM in relation to brief history of technology advancement, indications and currently available systems, published evidence on international practice and limitations), c) basics of new standardized glucose reporting system (rationale and utility, standardized CGM metrics, glucose statistics and targets, and analysis and interpretation of visual AGP data), and d) use of new standardized glucose reporting system in routine clinical practice (consensus recommendations and treatment and follow-up algorithm)

### Glycemic Parameters and Diabetes-Related Complications

Long-term hyperglycemia (reflected by elevated HbA1c), glycemic variability (inter-day variations in blood glucose), and glycemic instability (intra-day variations in blood glucose) are considered the major barriers to suboptimal glycemic control, while the risk of hypoglycemia is also increased by both glycemic variability and instability (5). Maintenance of adequate glycemic control is of critical value in the diabetes management, as associated with reduced risk of long-term morbidity and mortality (9, 10). The long-term hyperglycemia and glycemic variability are considered to increase the likelihood of diabetes related microvascular and macrovascular complications in T1DM and T2DM patients (11–13).

Additionally, while intensive glycemic control reduces the onset and severity of microvascular complications along with long-term cardiovascular benefits in T1DM and T2DM patients (14–16), hypoglycemia is also identified as a key determinant of increased risk of mortality in case of aggressively-targeted HbA1c (17, 18).

Hence, intensive glycemic control based on HbA1c targets without considering glycemic variability and instability profile is considered to be associated with increased risk of hypoglycemia, while glycemic variability rather than prolonged hyperglycemia is considered to be responsible for symptoms related to poor glycemic control (5, 18).

Thus, reducing glucose variability, accepted as a clinically valuable marker of glycemic control, is suggested to a valid therapeutic objective *per se*, alongside correction of elevated HbA1c (19–23).

Glycemic variability, in terms of both the amplitude and the timing of blood glucose fluctuations, has been associated with increased risk of hypoglycemia and hyperglycemia in diabetes patients (23, 24). This emphasizes the use of an accurate and standardized tool for glucose data collection and analysis that would reveal not only the overall glycemic patterns but also dynamic glycemic patterns and timing of deviations along with the hyperglycemic excursions (i.e., after meals) and potentially dangerous hypoglycemia (i.e., nocturnal) (1, 23, 25).

### Methods for Glucose Testing/Monitoring

While HbA1c, SMBG, and CGM are the three methods of testing glucose levels, the first two methodologies have been associated with significant drawbacks limiting their use in diabetes care (26).

HbA1c has been the key parameter in glycemic control assessment and the key surrogate marker for the development of long-term diabetes complications in T1DM and T2DM patients (7). However, given that HbA1c reflects the mean blood glucose over the life-span (~120 days) of red blood cells, it is not considered a good indicator of day-to-day diabetes control, glycemic variability, acute glycemic excursions, and associated risks of hypoglycemia or hyperglycemia (5, 7, 23, 25). The failure of HbA1c to reflect the diurnal glucose patterns is a major drawback considering the critical role of these patterns in making safe, effective, and timely insulin adjustment (1). In addition, HbA1c measurement is considered not reliable in certain confounding conditions such as pregnancy, hemoglobinopathies, anemia, and iron deficiency (7).

In contrast to HbA1c measurement, the use of CGM enables the direct observation of glycemic excursions and daily profiles and implementation of related therapy decisions and/or lifestyle modifications along with its ability to identify glucose variability and patterns of hypoglycemia and hyperglycemia (7, 23). Nonetheless, despite its limitations, HbA1c is the only prospectively evaluated parameter in assessment of the risk for diabetes-related complications, and thus should be used as a complementary method to CGM-based glycemic measurements (7).

SMBG, the intermittent finger-tip capillary sampling, is considered the gold standard, cheap, and readily available method for point-of-care glucose measurement (27). Although structured use of SMBG has been associated with improved glycemic control and quality of life (QoL) in diabetes patients (28, 29), it gives just snapshots of blood glucose concentration without capturing enough data points required to provide a complete story of daily glucose control, day-to-day glycemic variability and nocturnal and asymptomatic hypoglycemia and its application is dependent upon the patient’s decision to self-monitor (8, 23, 27, 30, 31). Thus, having limitations in detailed assessment of daily glucose fluctuations to guide the therapy for controlling the glycemic variability, using SMBG data *per se* may not reveal appropriate therapy decisions (5, 23).

Moreover, patient adherence to routine testing as per guidelines is very poor (only by 44% of T1DM patients and 24% of T2DM patients) due to factors such as fear of blood or needles, concerns about the frequency of application or perception of SMBG as a method used only for the insulin titration (32–35). Accordingly, demanding a self-testing strategy, getting reliable information *via* SMBG is rarely achieved in routine clinical practice despite proven efficacy in the research setting (5, 36).

However, CGM uses standardized metrics, glucose statistics, and targets to reflect the dynamics of glucose fluctuations and quantify glycemic variability and also hyperglycemic excursions (i.e., after meals) and potentially dangerous hypoglycemia (i.e., nocturnal) (1, 23, 25).

### Continuous Glucose Monitoring (CGM)

#### Brief History of Technology Advancements

CGM emerged by the new millennium as an innovative technology with potential to revolutionize diabetes care (37).

The major developments in the use of CGM in clinical practice included: a) the shift in CGM assessment from a retrospective to a prospective methodology to obtain real-time glucose readings by 2006, b) the identification of a research focus by its introduction to define the purpose of CGM (to characterize diurnal glucose patterns to detect abnormalities, to help identification and success of potential interventions for dysglycemia), c) the introduction of appropriate tools to optimize clinical decision-making by avoiding the errors that minimized the use of SBMG (i.e., incomplete understanding of the purpose, standardization accuracy, and reliability problems) and d) most recently the development of FGM systems that operate without necessitating calibrating interstitial glucose value to the capillary blood glucose and provision of AGP as a scientifically accurate and clinically reasonable method of reporting the dynamic properties of glucose metabolism (37–39).

Accordingly, CGM can provide both the real-time data on glucose levels and trends and the retrospective data on patterns of glycemic control over specified time periods and glucose metrics (1). Being a less-invasive approach than SMBG, CGM is considered to reveal an improved metabolic control with reduced HbA1c and/or the rate of hypoglycemia in T1DM and T2DM patients even in those already utilizing insulin pump therapy and those who have already achieved excellent control (1, 5, 30, 40).

The expert panel recommendations on CGM scope and technology are provided in **Box 1**.

Box 1 **Expert panel recommendations on CGM scope and technology.**

- CGM uses standardized metrics, glucose statistics and targets
- Reflects the dynamics of glucose fluctuations and quantify glycemic variability as well as hyperglycemic excursions (i.e. after meals) and potentially dangerous hypoglycemia (i.e. nocturnal)
- Inaccurate blood glucose readings, especially in the hypoglycemic range, and short sensor life have been barriers to the effective use of methods for the CGM previously
- Notably, the most recent advancements in CGM technology include introduction of isCGM systems that operate without necessitating calibrating interstitial glucose value to the capillary blood glucose and provision of AGP as a scientifically accurate, clinically meaningful method of reporting the dynamic properties of glucose metabolism

#### Indications for CGM

In diabetes care, CGM is used as both a short-term diagnostic tool (retroCGM, or professional CGM, sometimes blinded) and a long-term therapeutic tool (personal CGM) especially for T1DM patients (41), and in any patient on MDI or insulin pump therapy (42).

Indication of CGM is in accordance with its clear benefits regarding the clarification of glucose patterns and previously unknown hypoglycemic or hyperglycemic drifts, particularly for periods (i.e., nocturnal, postprandial) poorly explored by SMBG (41)

CGM is recommended to be used in combination with HbA1c in glycemic status assessment and therapy adjustment in all patients with poorly controlled T1DM or T2DM under intensive insulin therapy, particularly for those experiencing problematic hypoglycemia and/or have hypoglycemia unawareness (23, 42).

There are also miscellaneous indications for CGM with different levels of evidence which include the following (41):

- Brittle diabetes (variability analysis, assessment of potential causes such as premature needle withdrawal, intramuscular injection, and lipodystrophy)
- Flexible insulin therapy [FIT; facilitation of the assessment of FIT algorithms; in terms of basal insulin requirements (after fasting or a carbohydrate-free day test), carbohydrate ratios (for prandial rapid-acting insulin), and sensitivity index (for compensatory rapid-acting insulin)]
- Physical activity (glycemic effect of activity, validation of treatment options to avoid hypoglycemia during physical activity)
- Pregnancy (optimization of glycemic control)
- Discrepancy between HbA1c and SMBG (underestimation of HbA1c in dialysis patients, low/high hemoglobin glycation phenotypes)
- Certain clinical situations leading to variable glucose patterns (i.e., chronic dialysis, shift-work schedules, and defective compliance)

rtCGM devices are recommended to be used as close to daily as possible for maximal benefit, while FGM devices should be scanned frequently, at a minimum once every 8 h (42). CGM-based analysis involves a standardized report on metrics such as time in range, glycemic variability, patterns of hypoglycemia, and hyperglycemia (43). Glycemic variability data should also be considered in overall assessment of glycemic control, while the assessment of hypoglycemia should also include certain factors such as reduced awareness of subsequent hypoglycemia, cardiac arrhythmia, confusion, or abnormal or combative behavior, weight gain, and fear of hypoglycemia (23). Overall, entire CGM data should be evaluated within the context of other variables such as meals, treatments, exercise, illness, insulin boluses, and automated insulin delivery activity (43).

The expert panel recommendations on CGM indications in diabetes care are provided in **Box 2**.

Box 2 **Expert panel recommendations on CGM indications in diabetes care.**

- CGM refers to a short-term diagnostic tool (retroCGM, or professional CGM, sometimes blinded) or a long-term therapeutic tool (personal CGM) especially for T1DM patients
- Indications of CGM are in accordance with its clear benefits on identifying glucose patterns and previously unknown hypo- or hyperglycemic drifts, especially during periods poorly explored by SMBG such as night-time and postprandial periods
- CGM (rtCGM or isCGM) is recommended (in conjunction with HbA1c) for glycemic status assessment and therapy adjustment in all insulin-treated patients with T1DM or T2DM who are not achieving glucose targets, who are not meeting glycemic targets, have hypoglycemia unawareness, and/or have episodes of hypoglycemia
- CGM should be considered in all children and adolescents with T1DM, whether using injections or continuous subcutaneous insulin infusion, as an additional tool to help improve glucose control

#### Currently Available CGM Systems

Currently, the two CGM systems available are rtCGM and FGM [also called isCGM] (8). Both rtCGM [Dexcom G5 and G6 (Dexcom, Inc.) and Medtronic Enlite (Medtronic, Inc.), Eversense (Senseonics, Inc.)] and FGM [Freestyle Libre^®^ system (Abbott Diabetes Care, Alameda, CA)] sensors collect real-time glucose readings continuously, while there are certain differences between two systems (41) (**Table 1**).

**Table 1:** Basic features of rtCGM and FGM systems (41).

Differences	rtCGM	FGM
Real-time information	Automatically transmit the data to the reader or smartphone without user engagement every 5 min	The user must physically scan the sensor with a reader or smartphone at least once every 8 h to ensure optimal data collection, but the sensor measures the ISF glucose every minute
Data saving property	No, if connectivity is lost with receiver, data are also lost	Yes, saves a data point every 15 min
High/low glucose alerts	Yes	No
Connection with CSII pumps	Yes	No
Calibration	Once or twice daily with SMBG (Dexcom G6 can be calibrated with a scan code)	Factory calibrated
Wear life	5–10 days	14 days
sensor technology	Operate at higher electrical potential, low stability	Operate at a much lower electrical potential, improved stability
Insulin dosing decision	Not approved for users without SMBG test to confirm blood glucose levels (except for Dexcom G5 and G6 rtCGM systems)	Approved for insulin dosing without the need for an adjunct SMBG test
Interference from acetaminophen	Yes	No
Recommended sensor site	Abdomen (transcutaneous), upper arm (implantable)	Back of upper arm
**Common features**	**rtCGM and FGM**	
Directional trend arrows plus current glucose reading	Trend arrows provide information on the direction and the rate of change (RoC) of ISF glucose levels and are generated from the slope of ISF glucose values over the previous 15 min. The pairing of a current glucose reading with a directional trend arrow is a powerful tool to assist with making diabetes self-management decisions, not possible with SMBG testing	
Device specific reporting tools	AGP: provide data on collections of time-stamped glucose readings and trends over a single day, or many days	

rtCGM, Real-time continuous glucose monitoring; FGM, Flash glucose monitoring.

FGM is widely recognized as a convenient tool for cost effective glucose level monitoring with readings provided upon scanning of a sensor, an advantage for patients to obtain real-time glucose levels without the need to routinely run a finger prick test (30).

#### CGM in Routine Clinical Practice—Published Evidence

The use of rtCGM in adults and children with T1DM was reported to be associated with significantly reduced HbA1c levels (from 0.4 to 1.0%) in JDRF, DIAMOND, GOLD, and SWITCH studies (44–47), with improved TIR (1.3–2.3 h/day) in JRDF CGM, DIAMOND, SWITCH, IN CONTROL, and REPLACE-BG studies (44, 45, 47–49) and with reduced hypoglycemia risk in DIAMOND, GOLD, HypoDE, and CONTROL studies (45, 46, 48, 50), while a qualitative meta-analysis of rtCGM studies revealed that besides the established healthcare benefits of the method, rtCGM users experience certain physical, emotional, and social issues that should be properly addressed by education and support measures (51).

Referring to the newest technology, the use of FGM plus AGP system has generally been considered favorable with positive feedbacks regarding its ease of use and ability to capture information on glycemic variability and hypoglycemic episodes (2, 52). The FGM has important advantages such as an overall lower cost of acquisition and no need for patient calibration with SMBG, and the utility of FGM as an alternative to both SMBG and other methods of CGM is considered to rise substantially in the near future as a widely recognized convenient tool for a cost-effective blood glucose monitoring (30).

The following examples summarize the published evidence regarding the usefulness of the FGM in insulin-treated T1DM and T2DM patients. Overall, the reduction in HbA1c after using FGM is evidently demonstrated in many studies, particularly in patients with a suboptimal HbA1c and poor adherence to blood glucose monitoring rather than already motivated patients with well-controlled diabetes (30). Most studies revealed a statistically significant improvement in TIR, patients’ QoL and treatment satisfaction along with reduction of time spent in hypoglycemia and frequency of SMBG after the use of flash glucose monitoring, while the change in time spent in hyperglycemia was clinically insignificant (30).

##### Randomized Controlled Trials

- The IMPACT trial by Bolinder et al. in T1DM patients revealed 38% reduction in time in hypoglycemia and significant reduction in the mean number of SMBG (from 5.5 ± 2.0 to 0.5 ± 0.7) in FGM users (n = 119) as compared with the control (n = 120) group, while there was no significant change in HbA1c between both groups at 6-month follow up (53). There was no significant difference in Diabetes QoL (DQoL) score between both groups, while the Diabetes Treatment Satisfaction Questionnaire (DTSQ) score improved significantly in FGM users (53).
- In the REPLACE trial by Haak et al. covering T2DM patients, a 50% reduction in time in hypoglycemia and a significant reduction in the mean number of SMBG (from 3.9 ± 1.2 to 0.6 ± 1.2) were reported in FGM (n = 139) users vs. control (n = 62) group at 6-month follow up (54).
- The 8-week RCT by Reddy et al. in T1DM patients revealed the reduction in median HbA1c from 55 mmol/mol (159 mg/dl) to 51 mmol/mol (149 mg/dl) and the increase in median percentage time in hypoglycemia from 8.0% (IQR 5.7–10.7) to 8.2% (IQR 6.0–13.2) in FGM users (n = 20) (55).
- The 10-week RCT by Yaron et al. in T2DM patients showed significant reduction in HbA1c in FGM (n = 52) users vs. control (n = 44) group (−0.85% ± 0.45 vs. −0.32% ± 0.39), while no significant difference between groups in terms of frequency of hypoglycemic episodes (56). Mean DTSQ change (DTSQc) score was 2.47 ± 0.77 (FGM users) vs. 2.18 ± 0.83 (control) (p = 0.053). FGM users found it more flexible and would recommend to their counterparts. The Audit of Diabetes-Dependent QoL (ADDQoL) questionnaires scores were not significant between both groups (56).

##### Prospective Cohort Studies

- In a 12-month study by Paris et al. in 120 T1DM patients using flash glucose monitoring, significant change was noted in HbA1c from 70 mmol/mol ±1.5 (198 mg/dl ± 4.0) to 61 mmol/mol ± 10.4 (176 mg/dl ± 27.3), while the number of hypoglycemic events per month significantly increased from 16.9 ± 1.44 to 22.9 ± 2.03 (57).
- In a 3–6 month study by Heald et al. in 92 T1DM patients using flash glucose monitoring, significant change was noted in mean HbA1c from 83 mmol/mol (233 mg/dl) to 72.3 mmol/mol (205 mg/dl) at 3 months and 66.9 mmol/mol (191 mg/dl) at 6 months (58).
- In a 12-month study by Kramer et al. in 40 T1DM patients using flash glucose monitoring, no significant change was noted in HbA1c [from 57.6 mmol/mol ± 11.4 (166 mg/dl ± 29.9) to 57.1 mmol/mol ± 7.4 (165 mg/dl ± 19.4)], insulin dosing, number of insulin injections and BMI from baseline, while frequency of SMBG significantly decreased from 6.7 ± 4.2 to 0.9 ± 1.8 per day and DTSQc score increased by 12.6 ± 5.5 points (59).
- In a 6-month study by Overend et al. in 40 T1DM patients using flash glucose monitoring, absence of finger prick test was reported to be a major benefit with reduction in frequency and severity of hypoglycemia alongside good glycemic control and positive impact on psychological well-being and self-esteem (60).
- In a 6-month study by Tyndall et al. in T1DM patients, significant reduction was noted in median HbA1c (−4 mmol/mol (−10.5 mg/dl) from baseline) and median number of glucose test strip use per day (from 3.8 to 0.6), while percentage of patients with hospital anxiety and depression scale (HADS) depression (from 7.6 to 15.0%) and anxiety (from 24.9 to 30.9%) scores of >7 were increased from baseline in FGM users, and increase in median BMI was significantly higher in FGM users (n = 750, by 0.3 kg/m^2^) vs. control (n = 518, by 0.1 kg/m^2^) group (61).

##### Retrospective Cohort Studies

- In a 24-week study by Moreno-Fernandez et al. in T1DM patients, significant change in HbA1c (−0.4% vs. 0.1%) and decrease in SMBG per day (from 5.2 ± 2.5 to 2.8 ± 1.7) were noted in FGM users (n = 18) vs. control (n = 18) group with no significant difference between groups in frequency of hypoglycemic episodes (62)
- In a 3–12 months study by Nana et al. in 90 T1DM patients using flash glucose monitoring, significant change in mean HbA1c of −7.29 mmol/mol ± 10.76 (−19.1 mg/dl ± 28.2), 51.86% reduction in hypoglycemic episodes, significant reduction in frequency of SMBG per day and significant improvement in the abbreviated Diabetes Distress Scale (DDS) score were noted after FGM use (63).

Nonetheless, it should be noted that the currently available evidence on usefulness of FGM is drawn from T1DM and T2DM treated with insulin therapy and there is a need for further studies addressing the utility of FGM in non-insulin dependent T2DM patients. Also the impact on prevention of DKA or HHS has not been assessed in any of the studies (30).

In addition, data from a 6-month follow up study by Hermanns et al. indicated significantly improved HbA1c reduction, TIR, diabetes-related distress scores, and satisfaction with the glucose monitoring method among diabetes patients with vs. without participation in structured education and treatment program on FGM (64), while a prospective 12-month follow up study by Pintus et al. in T1DM children indicated significant improvement in patient QoL, reduction of diabetes symptoms, and treatment barriers after patients were trained in the use of the FGM system (65). Accordingly, patient education regarding FGM use is considered to provide an additional significant benefit regarding the reduction of HbA1c and also reduction of diabetes distress and enhanced satisfaction with glucose monitoring and better engagement in diabetes management, compared to the use of FGM technology alone (64, 65).

#### Current Limitations of rtCGM/FGM Systems

Most of the current devices on the market require finger-pricking *via* a standard home blood glucose monitoring system to confirm the glucose levels displayed on the CGM to be able to initiate the appropriate and most accurate intervention, which also raises another issue of not completely replacing the finger-pricking (66). However, while inaccurate blood glucose readings, especially in the hypoglycemic range, and short sensor life have been barriers to the effective use of methods for the CGM previously, introduction of longer-lasting, more accurate and cheaper sensors with improvements in sensor technology (FGM) eliminated the need for calibration measurements, as an innovative technology product (5).

However, there are potential drawbacks of rtCGM/FGM use (7, 8, 23, 66):

- Lag time (4 to 27 min, longer in adult vs. adolescent patients) when using a sensor due to physiological lag between interstitial fluid and blood and also the intrinsic effect of the device, greatly affecting the accuracy of the device and placing patients at risk for overdosing on insulin therapy or inadvertently inducing hypoglycemia. Nonetheless, newer algorithms have a shorter lag time.
- The risk of anxiety and consequent accuracy limitations due to requirement of the device to be actively used in order to be effective, particularly with the delay in registering blood glucose changes in dynamic situations,
- The risk of provoking skin allergies (devices like FGM have reduced the incident by removing allergens as IBOA from the sensor adhesive). The technology is not yet widely available in several regions of the world
- Requirement of adequate training on this new wave of technology by both practitioners and their patients to be able to use these medical devices both comfortably and effectively.

### Ambulatory Glucose Profile (AGP)

#### Rationale and Utility of AGP

Despite the benefits of CGM, the utilization of this technology in clinical practice has been suboptimal including only 3% of young T1DM patients (≤25 years) and 14% of older T1DM patients (26–49 years) (1, 67). The lack of software enabling relatively simple and standardized statistical and graphic visualization and interpretation of the glucose data has been a major contributor to the uncertainty and reluctance of clinicians to incorporate CGM into their practices (1, 3, 68, 69).

Thus, for many healthcare providers, the challenges of working with SMBG/CGM data have reinforced the practice of making therapeutic decisions by HbA1c values alone, despite its considerable limitations (69, 70).

Notably, recent improvements in monitoring technologies and establishing tools such as AGP provided a simple and informative method of analysis of the complex glucose data, and thereby a more consistent and standardized approach to the reporting and interpretation of this data in routine clinical practice (1, 8, 71, 72). Moreover, recommendations for the standardization of glucose reporting and analysis of continuous glucose data through use of the AGP were also published recently in order to optimize diabetes care (1, 5, 69).

Accordingly, AGP is currently recognized as an internationally agreed standard for summarizing and interpreting daily glycemic patterns using large amounts of data collected from rtCGM or FGM systems (1, 8, 72). By 2020, world-wide professional diabetes organizations recommended that the major manufacturers adopted the AGP as the primary visual means of representing CGM data for clinical decision-making within the standardized report (1, 8, 37, 42, 43, 73, 74).

The European consensus recommendations on the use of standardized glucose reporting system with AGP data in clinical practice suggest a week 4 review of the patient after the first assessment and then subsequent follow-up visits every 3 to 6 months (2).

The use of standardized glucose reporting system in combination with assessment of the patient’s daily routine and identification of times of day with increased risk of hypoglycemic or hyperglycemic events enables addressing potentially modifiable factors that are central to achieving good glycemic control in diabetes, and thus implementing specific changes to behavior and treatment (5, 25, 36). The AGP, representing the visual component of standardized glucose reporting system, is superior to glucose diaries for assessing hypoglycemic risk, while AGP readouts also provide a platform for constructive dialogue between members of the healthcare team and the patient, with potential for better engagement of patients in the management of diabetes and increased adherence to lifestyle intervention or changes to insulin or other pharmacotherapy (25, 36, 75).

The AGP displays large amounts of glucose data as if all the readings had occurred in a single 24-h period, while a profile can be created from at least 5 days to maximum of 3 months of such data (optimal period for reliability is 14 days) that provide important feedback on hypoglycemia and glucose variability along with information on the impact of insulin doses, meals, exercise, stress over single days or more-extended periods (8).

The AGP meets the three main purposes of CGM, namely, detection, intervention, and outcome (37) through providing data on glucose patterns to determine dysglycemia, to quantify glucose exposure, variability and stability, and to enable evidence-based clinical decision-making (37). Besides, by providing patterns *via* easily recognizable pictorial display, the use of AGP not only facilitates the analysis by the healthcare team and but also enables the patients with good self-management skills to easily identify and implement the necessary lifestyle of medication changes and thus changes the conversation between the patient and the healthcare team in the clinic (69). The AGP is documented to be a useful procedure for the analysis of glucose values in insulin-treated T1DM and T2DM patients, while evidence on its utility among insulin-naïve T2DM patients is lacking (3). The clinical situations in which AGP is considered useful (3, 25) are summarized in **Table 2**.

**Table 2:** Clinical utility of AGP (3, 25).

AGP	Clinical situations
**Useful in**	Comparison of the actual glucose values of the patient with the individual target values Analysis of the extent and causes of high glycemic variability Review of the suitability and appropriateness of a therapeutic strategy Testing the safety of adjusting a dose of insulin Clarifying the causes inconsistency in HbA1c and glucose profiles Recognizing asymptomatic hypoglycemia and hyperglycemia
**Less useful in**	Patients with poor compliance with treatment or with low motivation for changing behavior.

In fact, FGM is able to overcome SMBG’s limitation in glycemic variability detection and serve as a more affordable alternative with excellent accuracy to rtCGM without the need for calibration (30). In addition, the use of novel parameters in AGP analysis such as time in range and time spent in hypoglycemia that refer hyperglycemia or glycemic variability, respectively allow a more comprehensive overview of glycemic control than HbA1c and more informed treatment decisions (64). Thus, the use of AGP plus FGM is considered to represent the foremost innovative technology that has transformed diabetes care and had a positive impact on the psychological wellbeing in patients with diabetes that ultimately enhances patient compliance and ensures better glycemic control (30).

The expert panel recommendations on rationale and standardized reporting of AGP are provided in **Box 3**.

Box 3 **Expert panel recommendations on rationale and standardized reporting of AGP.**

- AGP overcomes the previous challenges of working with SMBG/CGM data and meets the need for software enabling relatively simple and standardized statistical and graphic visualization and interpretation of the glucose data to facilitate clinicians to incorporate CGM into their practices.
- Moreover, recommendations for the standardization of glucose reporting and analysis of continuous glucose data through use of the AGP were also published recently in order to optimize diabetes care.
- AGP provides a simple and informative method of analysis of the complex glucose data, and thereby a more consistent and standardized approach to the reporting and interpretation of this data in routine clinical practice.
- AGP-based visual assessment enables to summarize and interprete daily glycemic patterns using large amounts of data collected from rtCGM or FGM systems
- The European consensus recommendations on the use of AGP report in clinical practice suggest a week 4 review of the patient after the first AGP-based assessment and subsequent follow-up visits with analysis of AGP data every 3 to 6 months.

#### Key CGM Metrics: Visualization, Analysis and Documentation

Understanding and using the CGM generated glucose profiles and patterns are critical to managing diabetes and titrating therapy and is becoming easier given that CGM profile visualization is moving toward a standard AGP (1, 23, 76).

Effective use and appropriate interpretation of CGM data to optimize clinical outcomes is based on common metrics for assessment of glycemic status, graphical visualization of the glucose data and daily profile, and clear clinical targets (7).

##### Standardized CGM Metrics

The list of core standardized CGM metrics for the use in clinical practice recommended by the 2019 International Consensus statement (7) is provided in **Table 3**. The standardized CGM metrics include novel glucose statistics and targets such as time in range (TIR), time above range (TAR; high, very high, dangerously high), time below range (TBR; low, very low, dangerously low), and glucose management indicator (GMI) along with mean glucose and glycemic variability (7, 76).

**Table 3:** Standardized CGM metrics by the 2019 International Consensus recommendation (7).

STANDARDIZED CGM METRICS	
1. Number of days CGM worn (recommend 14 days)	
2. Percentage of time CGM is active (recommend 70% of data from 14 days)	
3. Mean glucose	
4. Glucose management indicator (GMI)	
5. Glycemic variability (%CV) target ≤36%	
6. Time above range (TAR): % of readings and time >250 mg/dl (>13.9 mmol/L)	Level 2
7. Time above range (TAR): % of readings and time 181–250 mg/dl (10.1–13.9 mmol/L)	Level 1
8. Time in range (TIR): % of readings and time 70–180 mg/dl (3.9–10.0 mmol/L)	In range
9. Time below range (TBR): % of readings and time 54–69 mg/dl (3.0–3.8 mmol/L)	Level 1
10. Time below range (TBR): % of readings and time <54 mg/dl (<3.0 mmol/L)	Level 2

**Figure 1:**
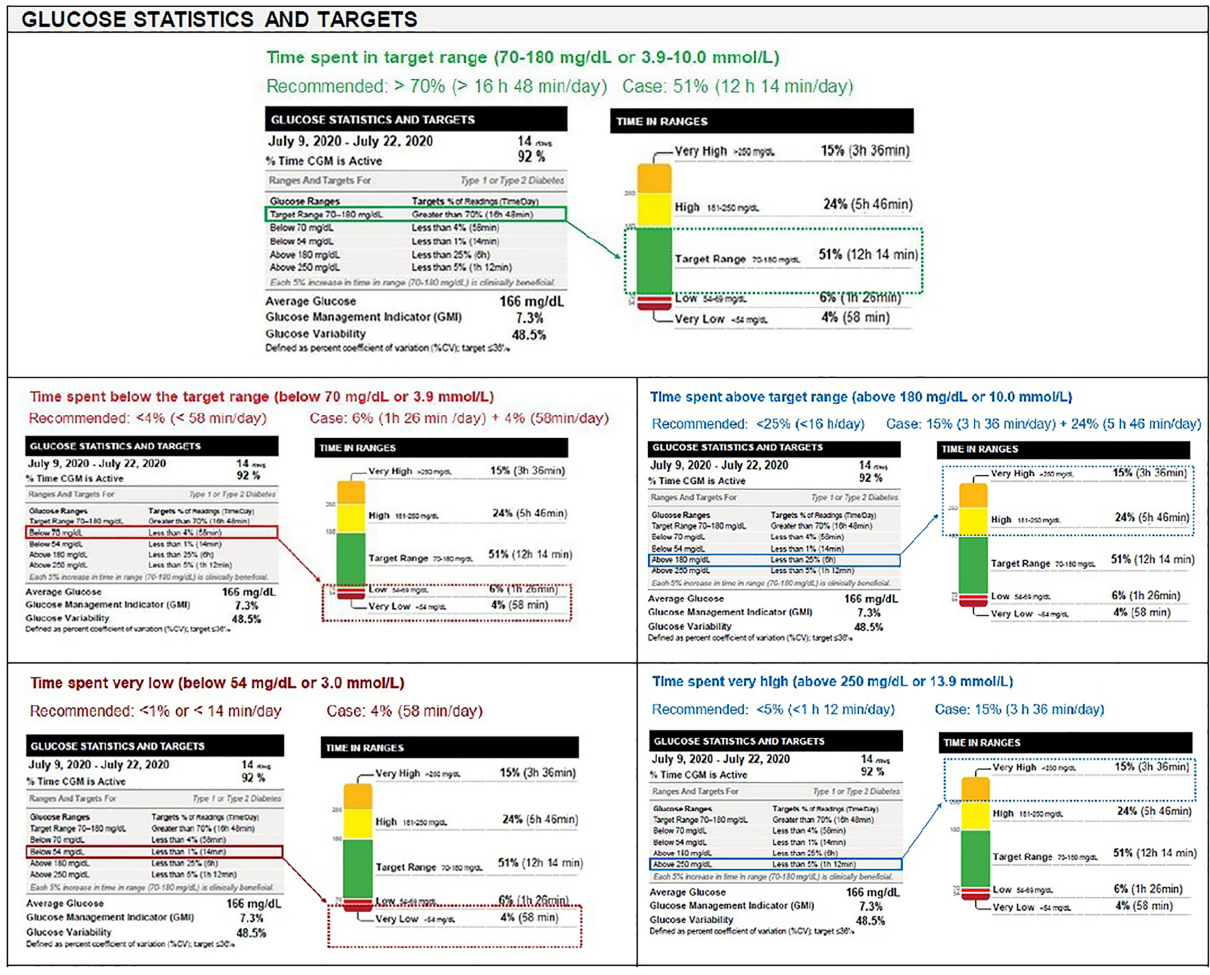
Glucose statistics and targets.

TIR refers to time spent in target glucose range (70–180 mg/dl, 3.9–10.0 mmol/L), while TBR [low (level 1): 54–69 mg/dl (3.0–3.8 mmol/L), very low (level 2): <54 mg/dl (<3.0 mmol/L)] and TAR [high (level 1): 181–250 mg/dl (10.1–13.9 mmol/L), very high (level 2): >250 mg/dl (>13.9 mmol/L)] are further categorized in two subgroups (Level 1 and Level 2) according to deviation from the target range (7) (**Table 3** and **Figure 1**).

##### Glucose Statistics and Targets

###### Time in Range

Recent consensus conferences (ADA, ATTD, and AACE) and publications have recognized that diabetes management needs to go beyond HbA1c and recommend aligning to common metrics on glycemic status including Time in Range (TIR) recommendations, standardization for AGP and daily glucose profiles, glucose Management Indicator (GMI) as a replacement for estimated A1c (eA1c), and is calculated using an updated equation, and clear clinical targets (7) (**Figure 1**).

TIR refers to one of the key metrics of CGM, providing more comprehensive information on glucose profile (short-term glycemic control) than HbA1c alone, and emerged as a novel metric for assessing glycemic control during recent years (77). TIR overcomes some of the inherent limitations of HbA1c besides its association with diabetes related complications (23, 77, 78).

A logical glycemic goal is thus considered to maximize TIR, while TIR alone is not an adequate description of overall glycemic control, it is also necessary to quantitate the times below (TBR) and above target range (TAR) (1, 23, 71, 79). Hence, a combined use of additional measures that quantify amount and severity of hypoglycemia and hyperglycemia is considered necessary to make TIR more broadly acceptable as a research end point or clinical measure (1, 23, 71, 79). TIRs are useful for a research comparison of interventions and can help patients understand whether the amount of clinically significant hypoglycemia or hyperglycemia they are experiencing is improving over time (4). Breaking out the time in hypoglycemia and hyperglycemia into level 1 (low and high, respectively; monitor and take action if needed) and level 2 (very low and very high, respectively; immediate action required due to the more potentially clinically significant nature of the glucose levels) can guide the urgency and degree of clinical response (23).

###### Glucose Management Indicator

Many CGM data reports include an estimate of A1C based on the CGM-measured mean glucose concentration, which might be closer, higher or lower than the actual laboratory-measured A1C (76, 80). This discordance between the eA1C (glucose in interstitial fluid) and the lab measured A1C (hemoglobin-attached glucose) can be confusing for patients and clinicians and the nomenclature of “eA1c” might imply there is a more direct relationship between the two (76). Accordingly, the term GMI, replacing the old term eA1C and calculated using an updated equation, is intended to convey that this is a measure calculated by converting CGM-derived mean glucose to a percentage and can provide an indication of the current state of a person’s glucose management (76).

**Figure 2:**
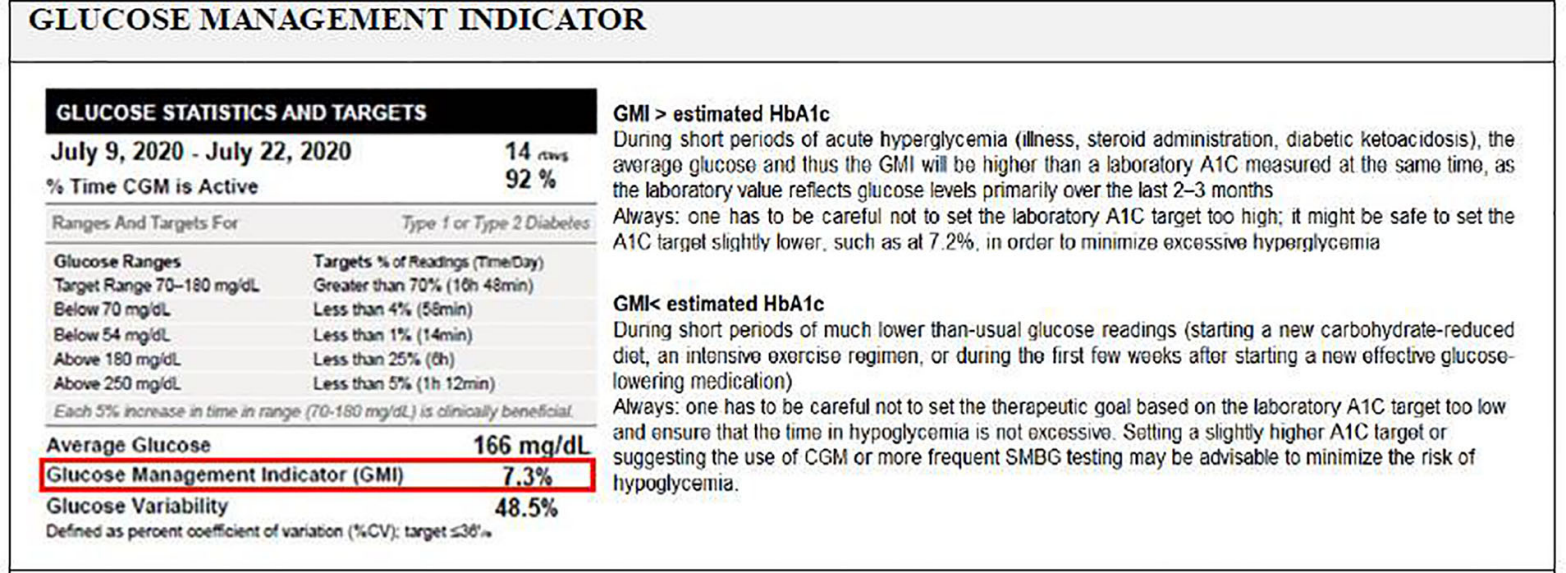
Glucose Management Indicator.

GMI can help patients and HCPs monitor progress but should not replace lab A1c tests (62). Differences between the GMI and laboratory measured A1C may reflect several conditions as summarized in **Figure 2** (76).

The expert panel recommendations on key points and clinical utility of AGP are provided in **Box 4**.

Box 4 **Expert panel recommendations on AGP- key points and clinical utility.**

- AGP is used in combination with assessment of the patient's daily routine and identification of times of day with increased risk of hypoglycemic or hyperglycemic events
- AGP and addresses potentially modifiable factors that are central to achieving good glycemic control in diabetes *via* providing important feedback on hypoglycemia and glucose variability and information on the impact of insulin doses, meals, exercise, stress over single days or more-extended periods (8)
- By providing patterns *via* easily recognizable pictorial display, AGP readouts also enables a platform for constructive dialogue between members of the healthcare team and the patient thus facilitates implementing specific changes to behavior and treatment.
- Accordingly AGP meets three main purposes of CGM including detection, intervention and outcome (37) through providing data on glucose patterns to determine dysglycemia, to quantity glucose exposure, variability and stability, and to enable evidence-based clinical decision-making,
- Use of novel parameters in the new standardized report including AGP analysis such as time in range and time spent in hypoglycemia that refer hyperglycemia or glycemic variability, respectively allow better overview of glycemic control than HbA1c and more informed treatment decisions
- Clinical utility of AGP involves comparison of the actual glucose values of the patient with the individual target values, analysis of the extent and causes of high glycemic variability review of the suitability and appropriateness of a therapeutic strategy testing the safety of adjusting a dose of insulin and clarifying the causes inconsisteney in HbA1c and glucose profiles

##### AGP Report

**Figure 3:**
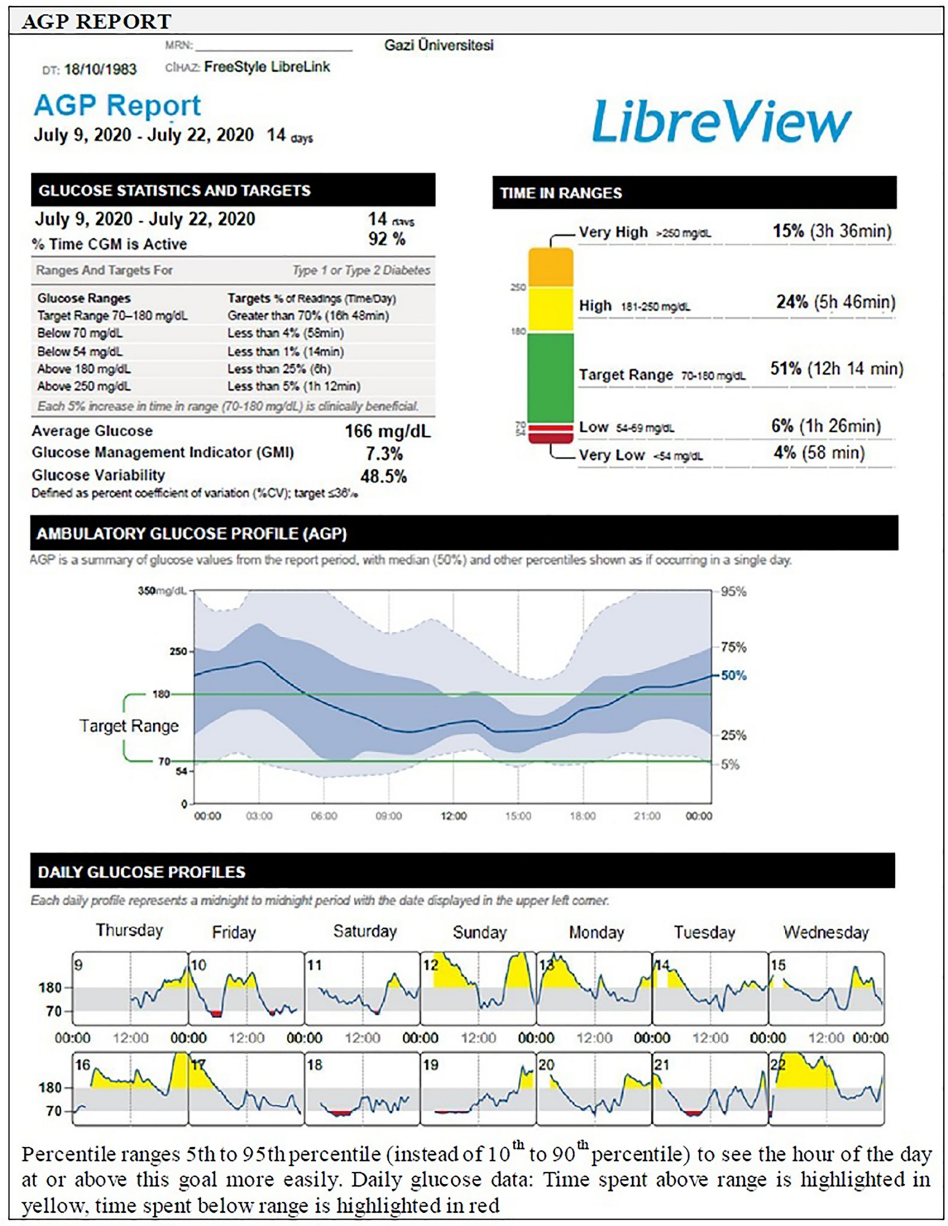
AGP report.

The AGP is a visual report and an easy to interpret graph that converts the readings obtained from CGM into a waveform based on pattern recognition, similar to an electrocardiogram. While the waveform will start to develop after at least 5 days of data collection, 14 days of data collection has been deemed ideal to most accurately reflect glucose control (1, 37, 39) (**Figure 3**).

Analyzing large amounts of glucose data from a number of separate days of recording collated in a single projection or a modal day, the AGP is presented as a median glucose value that reflects “what usually happens” rather than the mean which might be more strongly affected by outlying values, alongside the 25–75th and 10–90th percentiles (or 5–95th percentiles to see the hour of the day at or above the goal more easily), as calculated from the range of blood or interstitial glucose values at each time point (5). Interpretation is based on assessment of median, inter-quartile (IQR, 25th–75th percentile values) and inter-decile (IDR, 10th and 90th percentile or 5th to 95th percentile values) range curves that represent the central tendency and spread in glucose exposure, variability, and stability, over multiple days (7, 25, 37). The zone between the 25th and 75th percentile curves, accounts for 50% of all glucose values at any time point, the distance between the median blood glucose curve and those for the percentiles increases as the underlying glucose variability increases: the distance between the median and the 25th and 75th percentiles provides an indication of ‘usual’ glucose variability, while the 10th and 90th percentiles provide information on ‘occasional’ glucose excursions (7, 25, 37) (**Figure 3**).

Hence the AGP software, creates a standardized glucose reporting and analysis similar to electrocardiogram output and help the user to quickly identify areas of concern, namely, hypoglycemia or potential hypoglycemia, overall glucose control (TIR) and mean blood glucose value, and the degree of glycemic variability (5, 23, 69). A minimum of 14 consecutive days of data with approximately 70% of possible CGM readings over those 14 days appear to generate a report that has been validated as sufficient to provide a full analysis of issues relating to glycemic control in any given patient enabling optimal analysis and decision-making (5, 23) (**Figure 3**).

#### Expert Recommendations for the Use of AGP in Routine Clinical Practice in Turkey

Main obstacles of diabetes management today are the continued need for effective control of glycaemia, glucose variability and their relationship with diabetes complications, the continued need to limit the incidence of hypoglycemia; and these issues may be controlled by achieving a better understanding of daily glycemic control. Therefore, AGP might be particularly useful in managing patients who have poor exercise planning, who are doing active sports/swimming, who describe nocturnal hypoglycemia, hypoglycemia unawareness, fear of hypoglycemia and related suboptimal treatment adherence, who have pre-gestational diabetes, diabetes with high glycemic variability and/or morning hyperglycemia.

**Figure 4:**
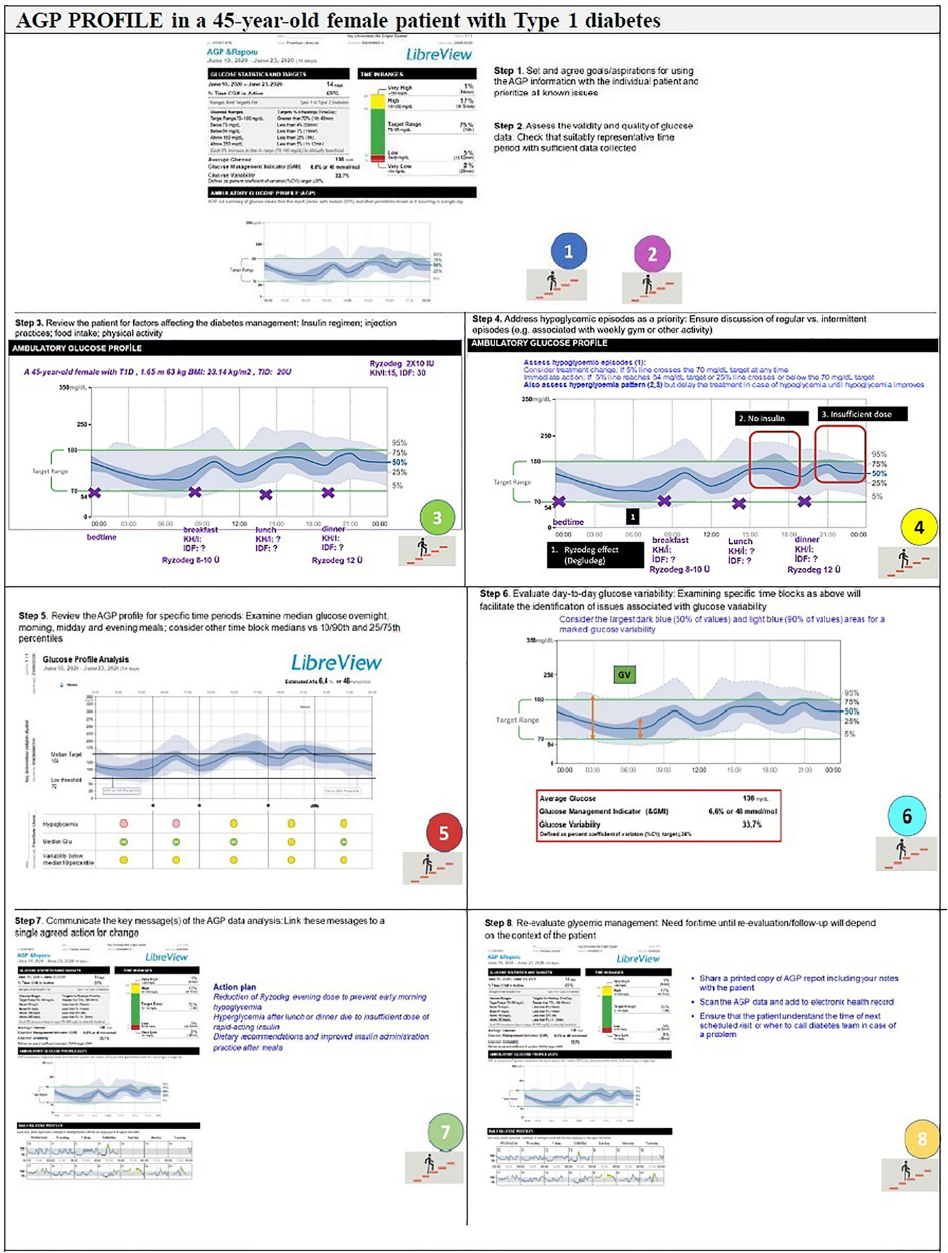
Recommendations for the use of AGP in clinical practice—Type 1 Diabetes.

**Figure 5:**
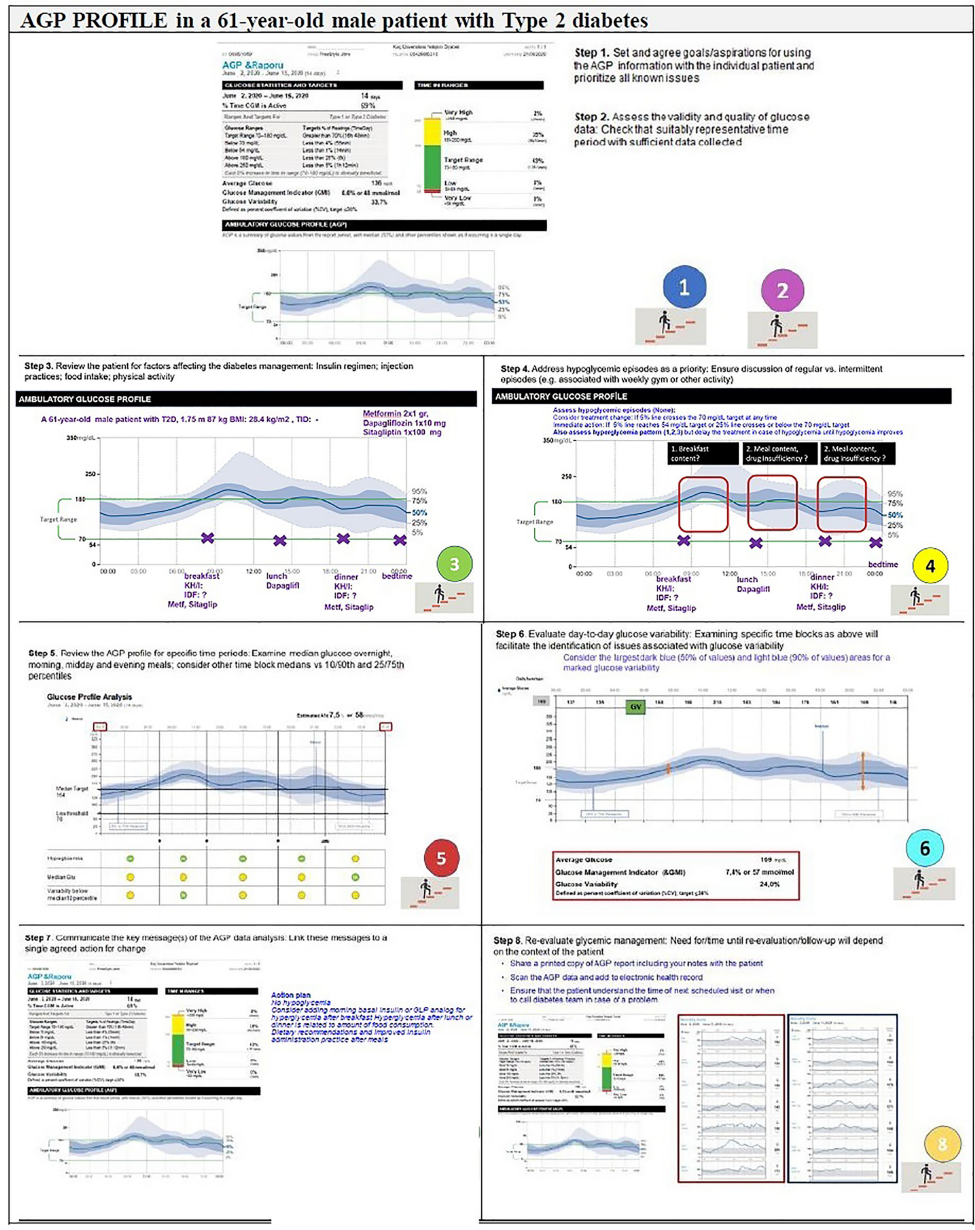
Recommendations for the use of AGP in clinical practice—Type 2 Diabetes.

The expert recommendations for the use of AGP in clinical practice include (**Figures 4**, **5**):

**Step 1.** Agreement on specific goals before starting the AGP analysis: Set and agree goals/aspirations for using the AGP information with the individual patient and prioritize all known issues

**Step 2.** Assess the validity and quality of glucose data: Check that suitably representative time period with sufficient data collected.

**Step 3.** Review the patient: Insulin regimen, injection practices, food intake, and physical activity.

**Step 4.** Address hypoglycemic episodes as a priority: Ensure discussion of regular vs. intermittent episodes (e.g. associated with weekly gym or other activity)

**Step 5.** Review the AGP profile for specific time periods: Examine median glucose overnight, morning, midday and evening meals; consider other time block medians vs 10/90th and 25/27th percentiles.

**Step 6.** Evaluate day-to-day glucose variability: Examining specific time blocks as above will facilitate the identification of issues associated with glucose variability.

**Step 7.** Communicate the key message(s) of the AGP data analysis: Link these messages to a single agreed action for change.

**Step 8.** Re-evaluate glycemic management: Need for/time until re-evaluation/follow-up will depend on the context of the patient.

**Figure 6:**
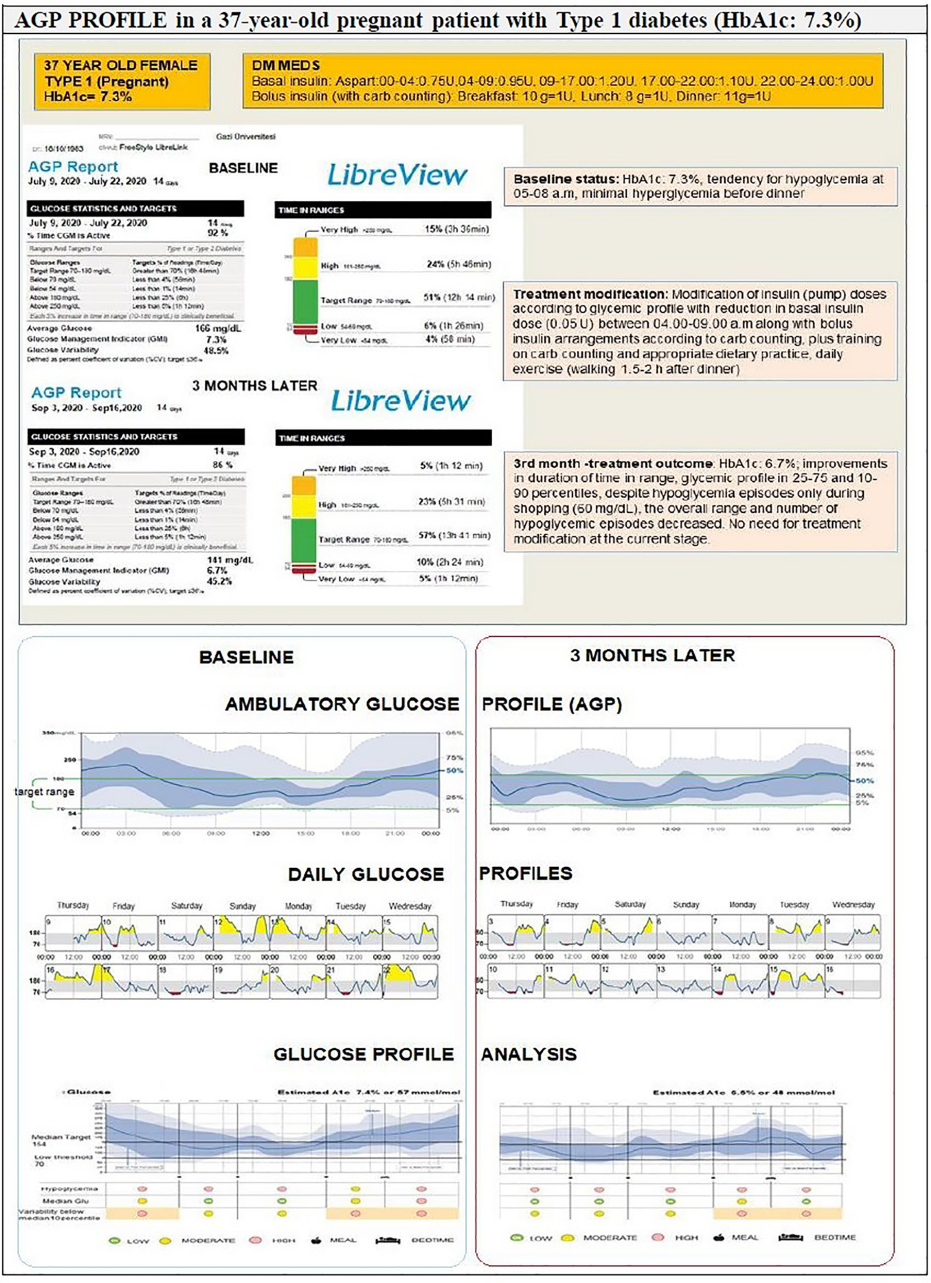
Example of using AGP in diabetes care: Treatment modifications and follow up outcome.

Overall, the clinical utility of AGP involves a comparison of the actual glucose values of the patient with the individual target values, an analysis of the extent and causes of high glycemic variability, a review of the suitability and appropriateness of a therapeutic strategy, testing the safety of adjusting a dose of insulin and clarifying the causes of inconsistency in HbA1c and glucose profiles (**Figure 6**).

## Conclusion

This paper prepared by an expert panel, provides a practical document to assist healthcare providers on the best practice with interpretation of a new standardized glucose reporting system based on standardized CGM metrics plus visual AGP data in diabetes care. Experts emphasize that the key factors supporting the clinical utility of standardized glucose reporting system are the comparison of actual versus target glucose values, detailed analysis of the glycemic variability, review of the appropriateness of a therapeutic strategy, test of the safety of adjusting insulin dosages and clarification of the inconsistency in HbA1c and glucose profiles, through an analysis of standardized CGM metrics and visual AGP data.

## Author Contributions

All authors listed have made a substantial, direct, and intellectual contribution to the work and approved it for publication.

## Funding

This expert panel study report was prepared with a workshop supported by the Abbott Diabetes Care Turkey which played a role in organization of expert panel meetings.

## Conflict of Interest

The authors declare that the research was conducted in the absence of any commercial or financial relationships that could be construed as a potential conflict of interest.

## Publisher’s Note

All claims expressed in this article are solely those of the authors and do not necessarily represent those of their affiliated organizations, or those of the publisher, the editors and the reviewers. Any product that may be evaluated in this article, or claim that may be made by its manufacturer, is not guaranteed or endorsed by the publisher.
